# The Immunological Microenvironment and the Emerging Role of Stem Cells Therapy in Peyronie’s Disease: A Systematic Narrative Review

**DOI:** 10.3390/ijms24010777

**Published:** 2023-01-01

**Authors:** Che-Hsueh Yang, Dian-Yu Lin, Yi-Sheng Lin, Chao-Yu Hsu, Min-Che Tung, Kok-Tong Tan, Yen-Chuan Ou

**Affiliations:** 1 Division of Urology, Department of Surgery, Tungs’ Taichung MetroHarbor Hospital, Taichung 435, Taiwan; 2 Joshua Taipei Hernia Center, Central Clinic & Hospital, Taipei 106, Taiwan; 3 Department of Urology, College of Medicine and Shu-Tien Urological Research Center, National Yang Ming Chiao Tung University, Taipei 106, Taiwan; 4 Division of General Surgery, Department of Surgery, Tungs’ Taichung MetroHarbor Hospital, Taichung 435, Taiwan

**Keywords:** erectile dysfunction, therapy, penile induration, penile diseases, physiopathology, penile diseases therapy, stem cell transplantation

## Abstract

Current literature has indicated that Peyronie’s disease (PD) could be initiated by microtrauma and the subsequent inflammation episodes that follow. PD could be sorted into acute or chronic status, and it can differ when selecting the clinical therapeutics. PD would cause pain and penile deformity to diseased men and impair their erectile function. Occasionally, surgical revision of the penis might be needed to correct the penile curvature. We find that there are limited effective options of intra-lesion injections for the PD plaques. By searching the databases and screening the literature with the PRISMA 2020 guideline, we observed that several preclinical studies that applied stem cell therapy in treating PD were fruitful in the acute phase. Although in the chronic phase of PD, erectile parameters were not significantly improved, and therefore, future studies might be better elevated in certain aspects, such as the sites selected for harvesting stem cells or changing the centrifugation forces. In this review, we concluded the contemporary understanding of inflammatory microenvironments in PD, the stem cell therapy in PD, and our perspectives on future studies. We concluded that there may be great potential in stem cell therapy for treating both acute and chronic phases PD.

## 1. Introduction

The prevalence of Peyronie’s disease (PD) in published literature has been seen to fluctuate in a wide range [1], from 0.32% to 11.8 % [2,3]. Using a validated questionnaire, the potential under-diagnosed cases were approximately fifteen times more than the diagnosed population [3]. Although the prevalence was hard to be estimated, several risk factors could be identified in the published literature, such as diabetes [1], β-blocker therapy [1], age [2], hypertension [4], smoking [4], and race [5]. The most indicative sign of PD was fibrotic plaques formation in either tunica albuginea (TA) or corpora cavernosa. However, the mechanism of PD is not fully understood. These plaques are hypothesized to arise from the abnormal healing process, making the stiff plates particle form inside the healing scars. As the plaques grow, they will lead to the pain and the deformity while erection. The consequences include impacts on the erectile function, self-esteem, and the psychological disorders [6,7]. Almost half of the men with PD experience significant impact on intimacy, and half of the men feel clinically meaningful depression [6].

Assessments of PD include a patient history such as symptom duration and progression, questionnaires such as a PD questionnaire [8,9] and International Index of Erectile Function [10], and a physical examination such as elasticity, penile length, and curvature, in addition to the plaques’ size and location. Without treatment, PD will not resolve by itself. Clinically, the options of treatment depend on the severity of PD. The options range from oral medication, topical agents, penile traction, shockwave, and intra-lesion injection. Aside from these treatments, when the curvature angle is greater than 60 degrees and there are risks of erectile dysfunction, surgeries such as prosthesis (with or without strengthening), for realigning the penile curvature can be considered. The practical considerations among various surgeries depend on the sophisticated assessments of the patients’ conditions, such as the penile length, deformity, and the evaluation from questionnaires [11].

Fibroblasts are physiologically responsible for supporting the organs by maintaining the homeostasis of the extracellular matrix (ECM) and its role is especially important when the tissues need repairing or remodeling. When the chronic inflammation exists, the inflammatory response will stimulate the accumulation of the proteins of the ECM’s connective tissues. In the modern understanding, the relations among tissues-resident mesenchymal stromal cells (MSC), mesenchymal progenitor cells, and fibroblasts have made researchers hypothesize that microenvironments might determine the specific roles of MSC in different organs, which harbor different cytokines and transduction pathways [12,13]. Moreover, some anti-fibrotic therapies are proposed based on this. Since the self-renewal and multipotent characteristics of stem cells (SC) make it more fascinating than other intra-lesion injection therapies, some well-known pathways and cytokines are investigated, such as the WNT pathway and Transforming Growth Factor Beta (TGF-β). Currently, exploring the therapeutic values of exosomes out of SC begins to bloom [14], and it has emerged as a new option in intra-lesion injection. 

## 2. Methods

**Figure 1:**
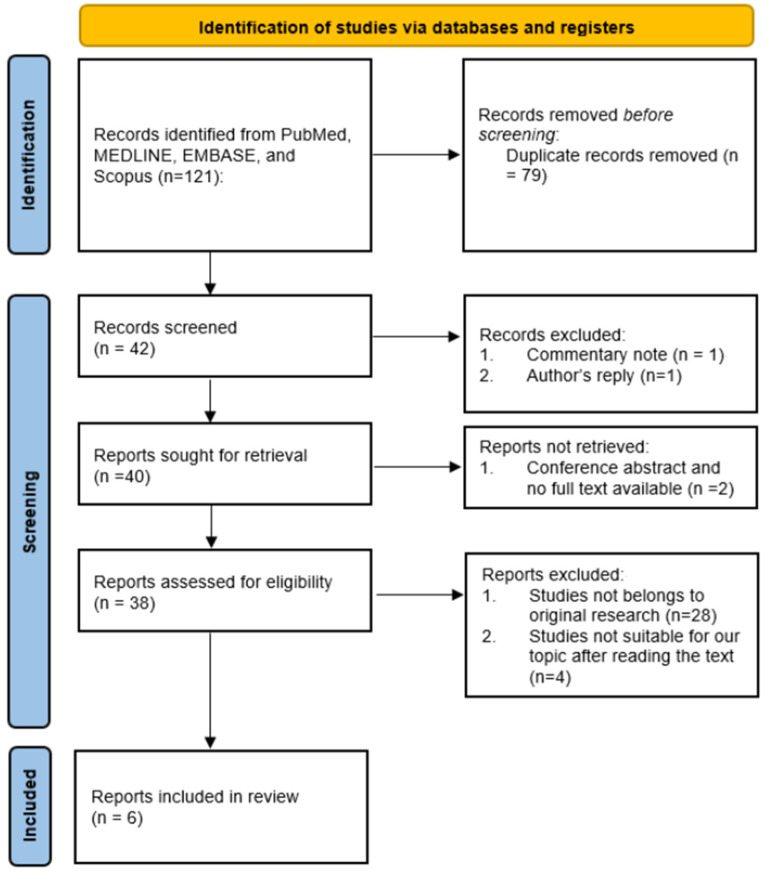
PRISMA flowchart in searching the original articles.

This review aims to draw the outline of the current application of SC in treating PD. The thesis is made based on the original articles searched on the PubMed, MEDLINE, EMBASE, and Scopus with terms such as “(Peyronie’s Disease) AND (Stem Cells)”. A total of 10 articles were retrieved [15,16,17,18,19,20,21,22,23,24]. Among them, 8 articles were categorized into basic science [15,16,17,20,21,22,23,24], while the other 2 articles were clinical studies [18,19]. After three urologists independently examined the texts, it was found that two of the articles in basic science [22,23] and one article in human study [19] were related to the stromal vascular fraction, while one article in basic science was related to the exosomes derived from human SC [24]. In this way, a total of six articles, one in human study and five in basic science study, were adopted for our main discussion (Figure 1). 

## 3. Results and Discussion

### 3.1. The Molecular Pathophysiology of PD

The fibrotic plaque of PD are a result of the excessive aggregation of fibronectin, elastin, and collagen in the penis. In this process, the fibroblasts will transform into α-smooth muscle actin (αSMA) -positive myofibroblasts [25], where the myofibroblasts are capable of secreting ECM contents and causing contracture and leading to the plaque and deformity of PD [26]. In PD, αSMA-positive myofibroblasts were found to be transformed from the fibroblasts in TA under the stimulation of TGF-β1 [27]. Thus, in the experiments studying the association between the genetic changes and PD, the most extensively studied one was the TGF-β1 gene. TGF-β1 was hypothesized not only to be responsible for the inflammatory response and the subsequent fibrotic changes in the process of PD, it could also be related to the progression of the disease [28]. Aside from this, microRNAs (miRNAs) are small endogenous non-coding RNAs, which could alter the expression of protein-coding RNAs. Among the microRNA-29 (miR-29) family, miR-29b is the one most often studied in interfering with the epithelial-mesenchymal transition (EMT) and the inhibiting fibrosis [29,30,31]. Meanwhile, there were also studies indicating that miR-29b could act as a regulator to TGF-β1 [32,33]. In a small case-control study, they found that the down-regulation of miR-29b was found in fibrotic plaques, TA, and corpus cavernosum in men with PD [34]. This result implied that the down-regulation of miR-29b and the subsequent up-regulation of TGF-β1 might be related to the pathogenesis of PD.

Aside from TGF-β1, the insulin-like growth factor-1 (IGF-1) could also be associated with the various conditions of EMT [35,36,37]. It could facilitate the wound healing process by activating fibroblasts and collagen synthesis. In men with PD, a study found that the IGF-1 was decreased in TA, and the isoform Ec was affected the greatest [38]. However, due to the limitations of statistical power, the TGF-β1 between men with and without PD was found to be not significant in this study. The possible type-2 error of this study had limited the conclusion regarding the correlation between IGF-1 and TGF-β1 in men with PD. Another large-scale study focused on the affects from the canonical WNT pathway. Since the pathology of PD seemed to be similar to Dupuytren’s disease, it was hypothesized that the two shared the common pathway in pathogenesis. In that study, they found the chromosome-7 WNT2 gene to be the most suspicious locus for PD [39]. This implied that the canonical WNT pathway might contribute to the PD’s fibromatosis.

### 3.2. The Immunological Microenvironment of PD

It is widely recognized that repetitive microtrauma to the penis and TA is the major cause of PD. This trauma theory has gained support from the animal model and the human plaque analysis [40,41]. However, this trauma theory only explained one of the many modifiable risk factors of PD, and not all men with such personal history would develop PD [42]. As such, exploring and expanding the understanding of PD’s molecular pathophysiology are essential [43,44].

Recently, immunological microenvironments in PD have been studied. In an animal study, it was found that one week after the injection of fibrin into TA, tissue edema would be observed, and it was believed to be the initial inflammatory response leading to PD [45]. Based on this study, fibrin could lead to more prominent early and late fibrosis than TGF-β1 alone. Interestingly, the final secreted TGF-β1 was more productive in rats injected with fibrin than in rats injected with TGF-β1. In assessing tissue inflammatory changes, the tissue edema would resolve when injecting fibrin and TGF-β1 at the same time. They hypothesized that since TGF-β1 was inhibitory to macrophages, the tissue inflammation by fibrin would be down-regulated when injected along with TGF-β1 [45].

When further examining the PD plaques induced from fibrin and TGF-β1, a more remarkable level of reactive oxygen species (ROS), myofibroblasts, and plasminogen activator inhibitors were observed in the PD plaques induced from the fibrin injection [45]. Aside from these, inducible nitric oxide synthase (iNOS) was observed to be elevated three weeks after injecting fibrin. This might be the response to the stress condition, such as inflammation, and would give rise to nitric oxide (NO) free radicals [46,47]. In spite of NO free radicals or ROS, they would increase the oxidative stress (OS) and eventually activate the Nuclear factor kappa-light-chain-enhancer of activated B cells (NF-κβ). Afterwards, NF-κβ would subsequently promote the pro-fibrosis-encoding genes, such as TGF-β1, fibrin, and iNOS and an excessive production of collagen would eventually ensue [48]. Recently, treating PD via the regulation of iNOS was proposed as one of the feasible modalities [49].

In real world experiences from a randomized controlled trial (RCT), oral propionyl-L-carnitine, targeting TGF-β1, and intra-lesion verapamil could remarkably reduce penile curvature, plaque size, cavernosal artery end-diastolic velocity. Furthermore, they could effectively slow the disease progression and decrease the progression to surgery. Patients after such therapy would also improve their subjective erectile function and the resistivity index of the cavernosal arteries [50]. Regarding antioxidants, a case report [51] showed that there was also great potential to applying antioxidants combined with therapy, which included oral (silymarin/Ginkgo biloba/propolis/bilberry/vitamin E) and topical (propolis cream) antioxidant in treating PD. In intra-lesion injections of antioxidants, RCT [52] revealed that the use of hyaluronic acid (HA) could effectively improve the penile curvature and allow patients a more satisfactory result than verapamil. In another RCT [53], intra-lesion verapamil and an oral antioxidant (vitamin E/para-aminobenzoic acid/propolis/blueberry anthocyanins/Soja isoflavones/Muira puama/Damiana/Persea americana) could more significantly enhance the patients’ subjective erectile function than intra-lesion verapamil alone.

**Figure 2:**
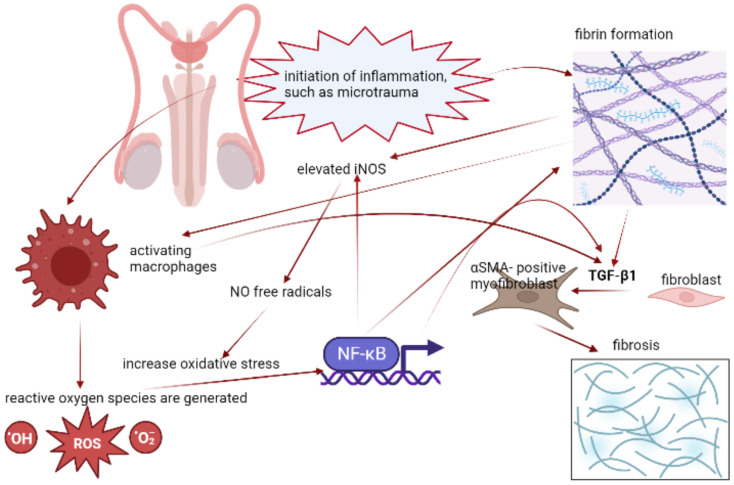
Inflammatory mechanism in PD. After the inflammation is activated, the activated macrophages and formation of fibrin will initiate the cascades up-regulating the fibroblasts transformation, and subsequently lead to fibrosis.

In summary, inflammation caused by fibrin would initiate the cascade of pro-fibrosis and down-regulate fibrolysis. Aside from these, it was postulated to have an innate mechanism to sustain this fibrotic loop and worsen the PD condition (Figure 2). Therapeutics interrupting this process could remarkably improve patients’ subjective erectile function and objective function index based on the evidence of RCT [50,52,53].

### 3.3. The Current Intra-Lesion Injection for PD

Although there are plenty of treatment options, no specific treatment is recognized as the best one. In clinical trials, assessing whether the patients are in the acute/early/inflammatory phase or the chronic/delayed/mature/quiescent phase is important for the subsequent treatments. PD plaques usually develop at the acute phase and are accompanied with pain, while the chronic phase features the stabilized deformity and the pain would completely resolve in most cases [54]. To distinguish between these two phases, an ultrasound and its related modalities are practical and useful [55,56]. In most cases, urologists would prescribe oral medications or intra-lesion injections to PD in the acute phase. The primary goals of such therapies include stabilizing the disease, reducing deformity, and improving sexual function. Once PD is stabilized, surgeries could be considered further.

Since the majority of patients with PD would seek medical help at their chronic phase, the current options of intra-lesion injection were sometimes not efficient. In the past forty years, around forty RCTs regarding medication treatments were published, and twenty-two of them were about the intra-lesion injection [50,52,53,57,58,59,60,61,62,63,64,65,66,67,68,69,70,71,72,73,74,75]. Currently, options of intra-lesion injection could be mainly categorized into calcium channel blockers (CCB), corticosteroids, interferon (IFN), and collagenase

#### 3.3.1. CCB

In CCB, options include verapamil [61,62,64,65,66,70] and nifedipine [71]. Intra-lesion injections of CCB could exert its therapeutic effect through inhibiting fibroblasts, increasing collagenase activity, deceasing collagen production, and modifying cytokines [61,62,66]. Some studies even mentioned that it could regulating the TGF-β1 activity [62,66]. It was also reported that it could be inhibiting interleukin (IL) 6, IL8, and plaque growth factor. As such, CCB would be able to down-regulate the inflammation initiating the fibrosis.

Intra-lesion injections of verapamil was safe according to the current literature of RCT, with side effects including dizziness, weakness, nausea, and sweating [61] but all of these required no further managements. However, not all patients and all conditions of PD could gain benefits from the intra-lesion injections of verapamil [66] and the ideal conditions included non-calcified plaque and angulations of less than 30 degrees [65]. After an injection of 10–27 mg of verapamil, the plaque size could be reduced by approximately 57%, and the penile curvature could be realigned by approximately 10 degrees. Aside from these, about 40% of men receiving such treatment could feel a subjective improvement on their erectile dysfunction [65]. Clinically, the standard dosage of verapamil was 10 mg in 10 mL of normal saline, and was injected every 2 weeks for 12-24 weeks. However, the data from the RCT revealed that 10 mg of verapamil in 20 mL of normal saline for 12 injections every 2 weeks could significantly improve the PD symptoms and signs such as pain, penile curvature, plaque size, and subjective erectile dysfunction [70]. Together with oral pentoxifylline, an antagonist against TGF-β1, the treatment effects would be further amplified [61].

Besides verapamil, another CCB, nifedipine, was also studied. The intra-lesion injection of 10 mg of nicardipine in 10 mL of distilled water after injections every 2 weeks for 6 times led PD symptoms and signs such as pain, plaque size, penile curvature, and subjective sexual scores to all be remarkably improved without any major side effects noticed [71].

#### 3.3.2. Corticosteroids

This treatment is straightforward in therapeutic theory, since it could down-regulate inflammation and prevent the plaque formation. However, corticosteroids possess the risk of tissue atrophy and destroying the penile tissue planes, and the treatment effect might not arise from the mechanical effect of injection but rather than the drug itself [76]. Thus, intra-lesion injection of corticosteroids was not recommended based on the current literature [77]. However, a recent clinical trial revealed that a weekly low-dose, 40 mg of methylprednisolone for 8 weeks could significantly downsize the plaques, thus improving the pain and subjective sexual satisfaction [78].

#### 3.3.3. IFN

IFNs are categorized to cytokines regulating the immune system. Among all kinds of INFs, IFN-α2β could inhibit fibroblasts proliferation, increase collagenase activity, and decrease collagen production. This way, it was thought to have therapeutic effects on treating PD. However, clinical trial did not support these supposed therapeutic effects [72], and only improvement on hemodynamics in Doppler ultrasound was reported [73].

#### 3.3.4. Collagenase

Its therapeutic theory is established on degrading the abnormal collagen deposits. Currently, the most studied type is collagenase from clostridium histolyticum (CH). The intra-lesion injection of CH collagenase could improve the PD symptoms [57,59,60,69,75] such as subjective sexual satisfaction [57], penile curvature [59,60,69,75] and plaque sizes [68]. Its therapeutic effects could last long after 5 years without additional treatments [59], and it was able to help those with penile curvatures of over 30 degrees, either alone [69] or in conjunction with a vacuum pump [60]. Generally, patients would be well-tolerated with it without significant side effects [74,75]. However, although CH collagenase could remarkably improve the symptoms and signs of PD, its availability due to the marketing strategy was another issue hampering it from widespread use [79].

Xiaflex (Endo International plc, Malvern, PA, USA) is the brand name of CH collagenase, and is the current and only intra-lesion injection medication approved by USA Food and Drug Administration. In guideline with the American Urological Association (AUA) [80], the intra-lesion injection of CH collagenase is on the highest evident basis among all intra-lesion options. According to AUA guidelines [80], it is not used in relieving painful plaques but is applied with the modeling protocol to reduce the penile curvature. The men indicated for this treatment that maintained intact erectile function a penile curvature between 30 and 90 degrees. The expected treatment effect on reducing penile curvature is described in Table 1 (IMPRESS trial [69]).

### 3.4. Examining SC Therapy in PD

The clinical considerations of PD and its current managements were summarized in Table 1. As mentioned above, the options for intra-lesion injections seem to be that CCB could not only offer certain benefits in improving PD symptoms and signs but also that its availability could be steadily supplied at the same time. However, prescribing the intra-lesion injection of CCB is on weak evidence based on the AUA guideline [80]. As such, developing new options for intra-lesion injections, whether in the acute or chronic phase, is an urgent issue. With the advanced knowledge about molecular biology and techniques regarding the cell’s cultivation, SC has had the potential of becoming the next injectable material with a promising future.

#### 3.4.1. The Current Animal Models for Establishing PD

As mentioned before, both fibrin and TGF-β1 have critical roles in the microenvironment of PD, including inflammation and fibrosis. The former would arouse more prominent early and later fibrosis than the latter [45]. Among the present studies, intra-tunical injections of TGF-β1 have remained to be the mainstream in establishing the animal models of PD [14,15,16,19,20] and the existence of this method can be traced back to approximately twenty-five years ago [81,82]. However, although these two methods were feasible theoretically, both of them had some deficits in nature. These deficits included expensive costs, high viscosity, and not being fully corresponded to the macroscopic changes of PD.

During the nature process of PD, the microtrauma to the penis would occur firstly. Secondly, the leak of fibrin would further release inflammatory cytokines, promote TGF-β1, and increase ROS production. Since the concentration of TGF-β1 was most abundantly enriched in plasma, injecting extracted plasma from vessels could not only reach the effected microtrauma but also give enough TGF-β1 to arouse the fibrotic process of PD. Aside from this, injecting self-extracted plasma from experimented animals would cost less than injecting synthetic fibrin or TGF-β1.

In the present animal studies regarding SC in treating PD [14,15,16,19,20], it established the PD induction model by injecting single synthetic TGF-β1. Currently, the PD parameters of erectile function included intracavernous pressure and tumescence slope, and the fibrotic assessments included collagen, elastin, tissue inhibitors of metalloproteinase (TIMP), and matrix metalloproteinases (MMP). However, although fibrosis was the center of pathophysiology of PD, other important clinical assessments consisted of pain and penile deformity caused by scar or fibrotic plaques. As such, the macroscopic parameters were still not included into the current preclinical studies of SC on treating PD. In one study [83], injecting four times the amount of plasma extracted from rats into a rats’ penis could arouse the effects similar to clinical changes of PD, including penile curvature and histological changes. The microtrauma by repeated injections and similar pathological results of this model could help discuss the macroscopic treatment effects of SC in treating PD. In mechanism, although there were proposed therapeutic values of regulating macrophages [84,85] and fruitful preclinical studies of applying SC in treating PD, the direct regulation of macrophages by SC were never demonstrated [14,15,16,19,20]. Judging from the other studies [86,87,88], this undiscussed mechanism of SC in PD is worth being experimented.

#### 3.4.2. Efficacy of SC in PD

In a preclinical study [14,19], SC could effectively improve the erectile and fibrotic parameters in the acute phase of PD. However, the treatment effect on erectile function would decline in treating the chronic phase PD [20]. In the only clinical study of ten men with PD, the intra-lesion injection to plaques was safe and no side effects were observed. Moreover, the size of plaques could achieve a complete remission in 70% of the plaques after 3 months’ post-injection, and it could also significantly improve the penile curvature [17]. However, the penile girth, stretched penile length, and subjective erectile function assessment were not remarkably recovered.

In clinical studies, a total of five men were enrolled, four of which were in the chronic phase. Herein, similar to the preclinical studies, the treatment effect of SC in improving erectile function seemed to decline in men with chronic phase PD [17]. There were no side effects observed in the study. However, this conclusion was too soon to be drawn, and a larger sample size of human trials should be conducted to discuss whether the erectile function improvement by SC treatment was limited in men with chronic phase PD. Aside from this, as the most effective and available intra-lesion injection options, CH collagenase and CCB were never discussed in comparison or combination with the application of SC in acute phase PD. Any preclinical and clinical results regarding this may be helpful to urologists and researchers to determine their strategies in treating men with PD or further discuss the efficacy of treatments.

#### 3.4.3. The Undiscussed Mechanism and Treatment Effect of SC in PD

The current animal and clinical studies regarding the SC therapies in treating PD were summarized in Table 2 and Table 3. Based on the present studies on the PD development and progression, the cascade of fibrosis could be aroused by initial trauma causing the subsequent inflammatory reaction. In the inflammatory process, the activation of macrophages was the core. The subsequent secretions of cytokines and chemokines would arouse the next fibrotic changes. In current studies [89], the polarization of macrophages allowed for dual roles in inflammatory and fibrosis to be played. The most known were the classical and alternative, which were also known as M1 and M2, respectively. They reacted to the different cytokines or chemokines, and consequently harbored the different characteristics. For example, M1 macrophages would react to the stimuli of toll-like receptors and interferon, and acted as the prime role in anti-parasite and anti-cancerous reactions. M2 macrophages would respond to the IL4/10/13 and TGF-β, and play important roles in the cancerous progression, as well as repair and remodel injured tissues. When the microenvironment was polarized to the M2 macrophages, the anti-inflammatory condition would be predominant. This macrophage polarization was observed in the fibrotic changes of several major organs, such as the kidneys, lungs, and liver [89]. For example, they found that the miRNAs could act as mediators to certain cytokines, such as NF-κB and TGF-β, to cause various effects on the macrophages polarization and result in renal fibrosis or nephropathy [90,91,92,93]. We previously mentioned that there was a study [34] indicating that down-regulated miR-29b was associated with the occurrence of PD in a case-control study, and it could be found that it was associated with regulating TGF-β1/Smad3 pathways [33]. Interestingly, down-regulated miR-29b was also found to be associated with renal fibrosis by affecting the macrophages polarization [89]. Although the role of macrophages in pathogenesis of PD was proposed [84] and was hypothesized to be of therapeutic value, this M1/M2 polarization was not clarified yet. A further investigation into the interaction among miR-29b, TGF-β1/Smad3, and M1/M2 polarization in PD may be of great scientific value to explore new medication. 

Regarding the treatment effect of SC on treating PD, the successful results could be seen in the acute phase of PD, however, the inferior results in treating chronic PD could be seen especially in the recovery of the erectile parameters. In current preclinical studies, all of them adopted adipose tissue-derived stem cells (ADSCs) in treating rats of TGF-β1-induced PD. Among various tissues cultivated for mesenchymal stem cells, adipose tissues could be easily acquired in large quantities with minimal invasive surgeries. In the preclinical studies we included, a total 3 [15,16,19] cultivated ADSCs from autologous fat. Although adipose tissues are widespread in human and animal bodies and potent treatment effects of ADSCs make them one of the most studied types of mesenchymal stem cells, there are still no standard harvesting procedures or protocols. In 3 preclinical studies from 2 different authors using autologous ADSCs they adopted different sites to harvest the adipose tissues and used different concentrations of type-1 collagenase, as well as different methods of centrifugation [15,16,19]. However, there were few studies comparing the differences on harvesting protocols in different studies. In one study comparing human ADSCs, it revealed that when considering surgical gain times and harvesting highly viable ADSCs cells, centrifugation with 400× *g* might be the efficient choice [94]. In examining the treatment effect of ADSCs in treating the chronic phase PD, centrifugation with 1000× *g* was adopted [14,20,95]. However, this might affect the viable cells under such centrifugation force, and further affect the results of the treatment effect on remedying chronic PD. In future studies designed for investigating the treatment effect of ADSCs on chronic PD, a better result by increasing the viable cells could be anticipated when choosing 400× *g*.

Aside from this, adipose tissues could be mainly categorized into white and brown. White adipose tissues, residing mostly in subcutaneous and visceral fat, were associated with the storage of excessive energies, while brown adipose, residing mostly in mediastinum and neck tissues, were related to the thermogenesis [96]. The sites selected for harvesting adipose tissues for cultivating ADSCs could be important. In human bodies, the ADSCs from subcutaneous and visceral fat featured different surface markers [97], and commonly the former was thought to be more abundant than the latter [98]. Moreover, among the different sites of white fats harvested for ADSCs, some distinctions would exist. Subcutaneous inguinal tissues could be different from visceral epididymal adipose tissues. In function, there was a study revealing that CXCL14+ ADSCs from subcutaneous inguinal tissues would feature suppressing macrophages infiltration, while SDC1+ ADSCs from visceral epididymal adipose tissues would feature modulating fibrotic changes [99]. In treating chronic PD, fibrosis might be the primary issue to cope with. In this regard, changing the different type of ADSCs could be beneficial, and the cultivation of SDC1+ ADSCs from visceral epididymal adipose tissues might be better in treating chronic PD.

**Figure 3:**
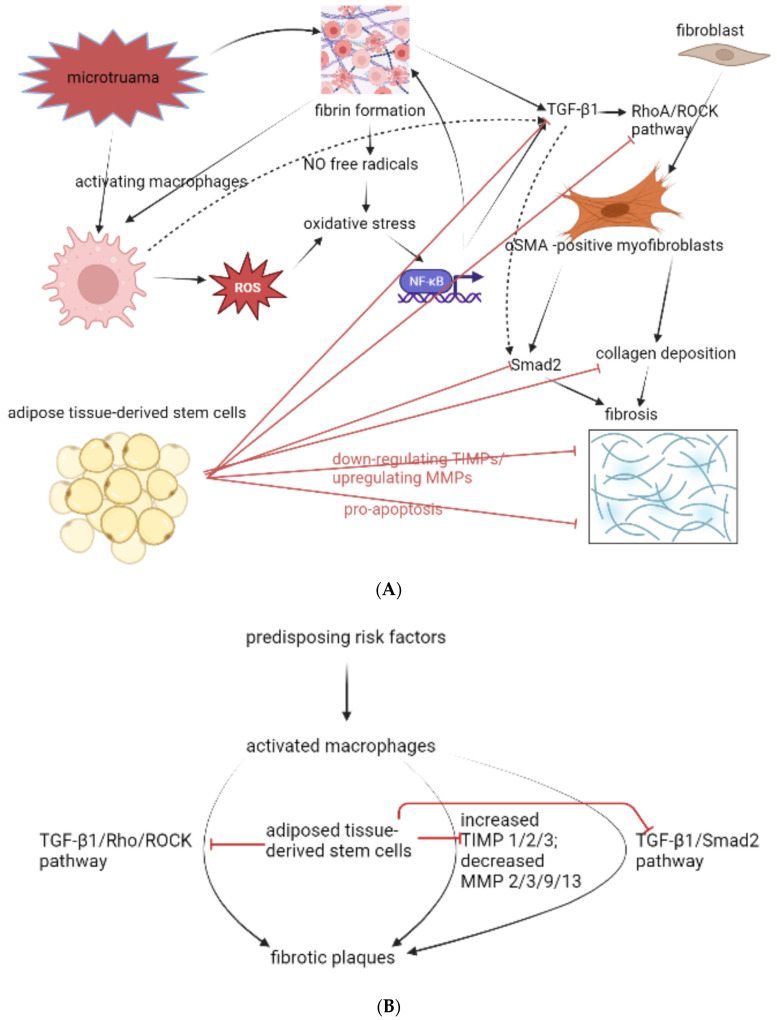
(**A**) The current preclinical studies of applying ADSCs in treating PD. In published literature, only the downstream pathways were discussed. Several pivotal impacts by ADSCs were not yet addressed, such as macrophages polarization, free radicals, oxidative stress, and NF-κB. (**B**) The abbreviated illustration of how ADSCs alter the immunological microenvironment of PD. In the published preclinical studies, the most mentioned immunological microenvironments changes by ADSCs were inhibiting TGF-β1/Rho/ROCK, and inhibiting TGF-β1/Smad2. Moreover, the increased TIMPs and decreased MMPs were reversed by the ADSCs.

Current preclinical studies regarding the application of ADSCs in treating PD were fruitful (Figure 3). However, the anti-fibrotic mechanism in the current preclinical studies seemed to be concentrated on the inhibiting downstream signal pathways, such as TGF-β1/Rho/ROCK and TGF-β1/Smad2, and adjusting imbalanced TIMPs/ MMPs. These strategies were also effective in reversing fibrotic changes in other organs [100,101,102,103]. However, when the emerging role of activated macrophages in pathogenesis of PD was receiving attention, there were still no studies discussing the inhibition of macrophages by ADSCs. As an upstream initiation to the subsequent cytokines cascade and pathogenic signal pathways, being able to inhibit activated macrophages might highlight ADSCs as a more powerful weapon than the current intra-lesion injection options treating PD, such as CCB. In a preclinical study using human ADSCs in treating rats’ acute kidney injury [104], ADSCs exerted capabilities inhibiting macrophages infiltration and decreasing OS. These two pathogeneses might also be the early initiation in the pathogenic inflammatory microenvironment of PD. Herein, exploring the relations among ADSCs, infiltrating macrophages, macrophages polarization, and OS would allow for SC therapy in PD to be more fulfilled and comprehensive.

## 4. Conclusions

The present preclinical and clinical studies revealed that ADSCs could be effective in treating the acute phase PD and be safe in applying it to the human bodies. However, some erectile parameters were not significantly improved in the preclinical and clinical studies in the chronic phase PD. Currently, SC therapies in treating erectile dysfunction are rapidly blooming, and they are reported as safe with good efficacy [105]. Since the evaluation of erectile function is also one of the considerations when choosing a treatment in the AUA guideline, restoring men’s erectile function from PD may be another promising future of SC therapies in treating PD. With more understandings to the ADSCs cultivated from white fats, changing experimented ADSCs might be another solution to examine the treatment effect of ADSCs in treating chronic phase PD. Simultaneously, with the emerging role of macrophages and its subsequent signal cascades and pathways, the undiscussed mechanism of ADSCs could be further fulfilled and become more comprehensive than the current available options of intra-lesion injection of PD.

## Figures and Tables

**Table 1:** The summary of PD and its current treatment options. ^a^ Computed tomography; ^b^ Magnetic resonance imaging; ^c^ Collagenase Clostridium Histolyticum; ^d^ Calcium channel blocker; ^e^ Interferon.＊ The treatment effect of intra-lesion injection of CCH ^c^, compared with the placebo group, on penile curvature is evaluated with 2 injections of 0.58 mg CCH ^c^; the second injection was given at 24 to 72 h after the second injection, and the treatment effect was compared between the baseline to the data of 52 weeks after intra-lesion injection.

Key factors	Genetics Trauma Ischemic changes
Clinical symptoms and signs	Painful penile plaques Penile deformity or curvature Erectile dysfunction
Clinical stages	Acute phase: painful penile plaque, progression of penile curvature, or deformity Chronic phase: absence of painful penile plaques, non-progression of curvature, or deformity
Image tools for assessment	Ultrasound (priority choice) Plain X-ray CT ^a^ MRI ^b^
Therapeutic methods	Observation: chronic phase and curvature ≤ 30 degrees with preserved erectile function Medical treatment (including intra-lesion injection): progression of penile curvature and unsatisfactory erectile function Surgical treatment: severe penile deformity or curvature, such as hourglass shape; erectile dysfunction refractory to medical/intra-lesion treatment; large penile plaques
Types of intra-lesion injection	Collagenase: CCH ^c^ is the only intra-lesion choice approved by the US Food and Drug Administration CCB ^d^ IFNs ^e^ Corticosteroids
Study endpoints in clinical studies	Penile curvature or deformity Penile pain Sexual life Size of plaques
＊ Curvature improvement after intra-lesion injection CCH ^c^ [69]	Categorized by curvature degrees: 30–60 degrees: reduction by 14.8 degrees (33.8% of the original curvature) 60–90 degrees: reduction by 25.3 degrees (35% of the original curvature) Categorized by PD duration: 1–2 years: no significant improvement of penile curvature 2–4 years: reduction by 17.3 degrees (33.8 of the original curvature) 4 years: reduction by 19.5 degrees (39.6% of the original curvature)

**Table 2:** The current animal and *in-vitro* studies of stem cells therapy in Peyronie’s disease (PD).

Study	Purpose	SC Isolation	Treatment Courses	Treatment Effect
*Castiglione et al. [15]*	Acute phase †	ADSC was cultivated from the subcutaneous adipose tissue from a female adult human	Inducing PD by intra-tunical injection of TGF-β1 Intra-tunical injection of ADSC on day 1 after the PD induction	Erectile function improvement: ICP and ICP/MAP Tumescence slope Fibrosis and elastosis improvement: Elastin: decreased by 47.7% Collagen III: decreased by 69.9%
*Gokce et al. [16]*	Prevention and treatment ‡	ADSC was cultivated in the inguinal fat tissue of SD rats	Inducing PD by intra-tunical injection of TGF-β1 Intra-tunical injection of ADSC either simultaneously or at day 30 after PD induction	Erectile function improvement: ICP/MAP (after treatment and prevention) Fibrosis and elastosis improvement: Decreased fibroelastic changes (after treatment and prevention) Decreased TIMP 1/2/3 expression (only after prevention) Increased MMP 1/2/3/9 expression (after treatment and prevention)
*Gokce et al. [17]*	Prevention and treatment ‡	ADSC was cultivated in the inguinal fat tissue of SD rats Human IFN α2b	Inducing PD by intra-tunical injection of TGF-β1 Intra-tunical injection of ADSC was either simultaneous or at day 30 after PD induction ADSC alone or in combination with human IFN α2b	Erectile function improvement: ICP (after preventive ADSC and treatment ADSC-IFN α2b) Combination with human IFN α2b: more significantly improved ICP than ADSC alone ICP/MAP (only after preventive ADSC) Fibrosis and elastosis improvement: Decreased fibroelastic changes (after treatment and prevention; ADSC alone or in combination with human IFN α2b) Decreased TIMP 1/2/3 expression (only after treatment ADSC-IFN α2b) Increased MMP 1/9 expression (after treatment and prevention; ADSC alone or in combination with human IFN α2b) Increased MMP 2/3 expression (only after preventive ADSC-IFN α2b)
*Jiang et al. [20]*	Discussing the *in-vitro* pathophysiology	ADSC was cultivated from the paratesticular adipose tissues of SD rats	αSMA-myofibroblast: treating fibroblasts with TGF-β1	Fibrosis, elastosis, and apoptosis improvement: Decreased αSMA-myofibroblasts growth Decreased secreted collagen Decreased collagen I Decreased phosphorylated Smad2 Decreased RhoA, ROCK1, and ROCK2 Increased MMP 2/3/9/13 expression Increased cleaved caspase 3, caspase 9, and cleaved caspase 9
*Castiglione et al. [21]*	Chronic phase †	Human ADSC was cultivated from the human female subcutaneous adipose tissue	Inducing PD by TGF-β1 intra-tunical injection of TGF-β1 Intra-tunical injection of ADSC at 1 month after PD induction	Erectile function improvement: No significant changes in ICP No significant changes in ICP/MAP Fibrosis and elastosis improvement: Decreased Collagen III/I ratio Decreased CCL13, CXCR4, and TGF-β1

† The acute phase and the chronic phase were determined by the time interval between the PD induction and ADSC injection. ‡ The prevention was that ADSC was injected at the same day as the PD induction; The treatment was that ADSC was injected at 30 days after PD induction. SC: Stem cells; ADSC: adipose tissue-derived stem cells; PD: Peyronie’s Disease; ICP: intracavernous pressure; MAP: mean arterial pressure; SD: Sprague-Dawley; TIMP: tissue inhibitors of metalloproteinase; MMP: matrix metalloproteinases; IFN: interferon; αSMA: α-smooth muscle acti.

**Table 3:** Conclusion of the current human clinical studies of SC in PD.

Study	Case	Dose and Injection	Significant Improvement	Insignificance
*Levy et al. [17]*	Enrollment: 5 men; 45–59 years’ old 1 man in acute phase; 4 men in chronic phase Number of plaques: 10	Placental matrix–derived MSC (abstracted from chorionic placenta) Injection: at the plaques and both corpora	Plaque size Penile curvature Erectile function: peak systolic velocity	Penile girth Stretched penile length Erectile function: End-diastolic velocity International Index of Erectile Function questionnaire

MSC: mesenchymal stem cells.

## Data Availability

Not applicable.
